# Geodynamic models of Indian continental flat slab subduction with implications for the topography of the Himalaya-Tibet region

**DOI:** 10.1038/s41598-024-52709-w

**Published:** 2024-01-29

**Authors:** K. Xue, W. P. Schellart, V. Strak

**Affiliations:** Department of Earth Sciences, Vrije Universiteit Amsterdam, Amsterdam, The Netherlands

**Keywords:** Geodynamics, Tectonics

## Abstract

The slab structure and high elevation of the Himalaya-Tibet region and their underlying mechanisms have been widely discussed. Many studies interpret a flat slab segment of Indian continental lithosphere located below the overriding plate, but interpretations of the northward extent of the flat slab differ substantially, with minimum estimates placing the boundary at the northern margin of the Himalaya (Indus-Yarlung Tsangpo suture), and maximum estimates placing it at the northern boundary of Tibet. In this study, we investigate for the first time if a flat slab segment of subducted buoyant Indian continental lithosphere below the Himalaya-Tibet region is geodynamically feasible and we quantify its northward extent, as well as its contribution to the high topography of the region. We conduct three large-scale fully-dynamic (buoyancy-driven) analogue experiments to simulate the subduction of the Indian continent. Our preferred, and geodynamically most feasible, model shows a continental flat slab extending northward up to ~ 320 km from the Himalayan thrust front, in agreement with recent estimates. Furthermore, it suggests that the positively buoyant flat slab segment of the Indian continent contributes some ~ 1.5–2 km to the high topography of the Himalaya-Southern Tibet region by providing an upward force to elevate the overriding Eurasian plate.

## Introduction

**Figure 1:**
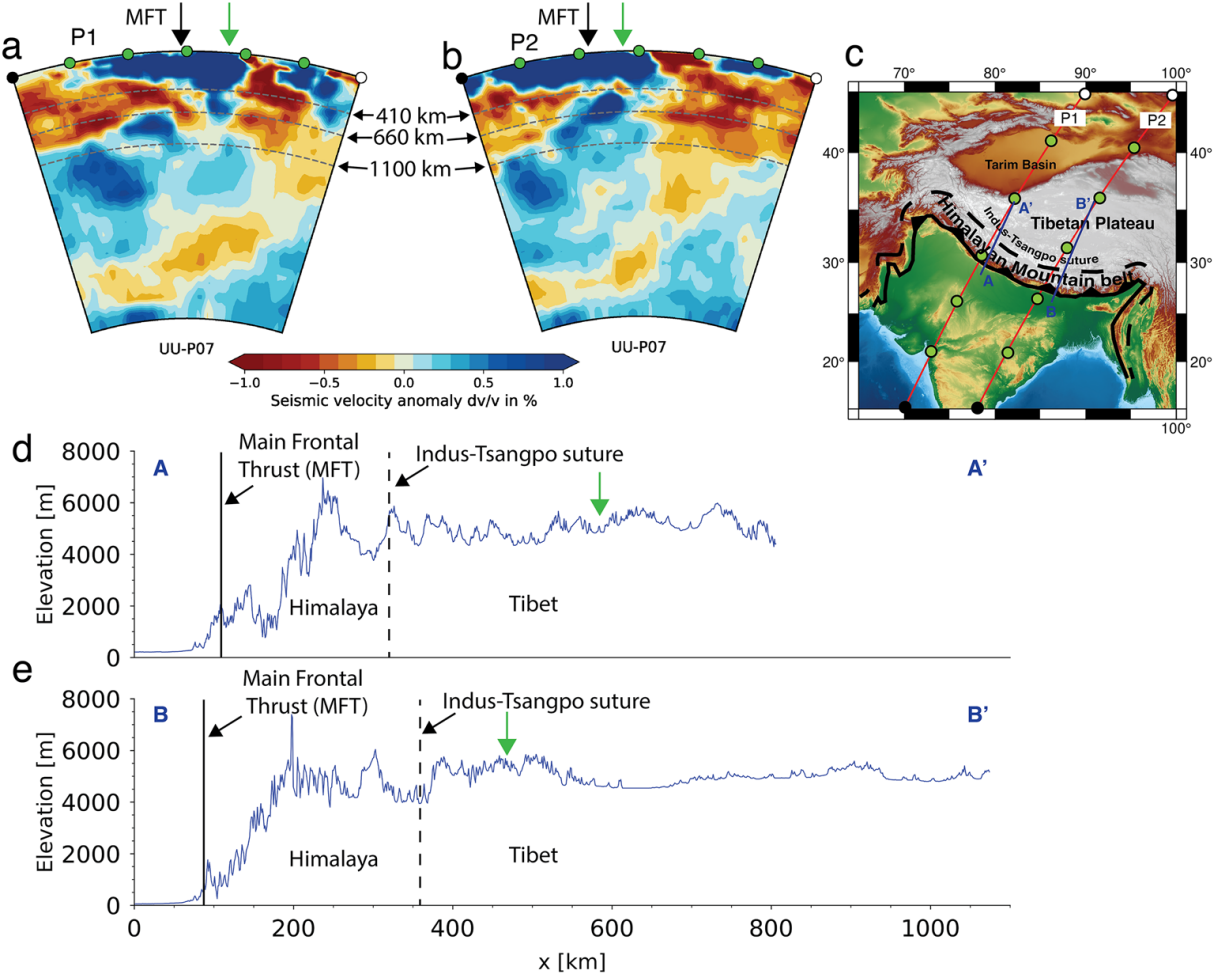
(**a**,**b**) Seismic tomography across the India-Eurasia collision zone from the global P-wave seismic tomography model of Amaru^32^. (**c**) Topographic map of the India-Himalaya-Tibet region illustrating the location of the tomography profiles (P1 and P2) shown in (**a**) and (**b**) and the topography profiles (AA’ and BB’) shown in (**d**) and (**e**). (**d**,**e**) topographic profiles across the India-Eurasia collision zone. Green arrows indicate the approximate downdip limit of the Indian continental flat slab as implied by the seismic tomography sections in panels (**a**) and (**b**).

The Himalaya-Tibet mountain system in Asia, the highest mountain range in the world, has an average elevation of ~ 5 km (Fig. 1)^1^, formed mainly due to tectonic processes since the India-Eurasia continent–continent collision started at ~ 50 Ma^2–9^. The Himalayan mountain belt extends from the India-Eurasia collision zone plate boundary northward over a distance of ~ 200–320 km, until the southern margin of Tibet (Indus-Yarlung Tsangpo suture), while the Tibetan plateau extends northward over a distance of ~ 1000 km. Different conceptual models have been proposed to explain the formation of the high topography of the Himalaya-Tibet region, such as the underthrusting model^6,10–13^, the crustal thickening model through localized^14,15^ or distributed deformation^16,17^, and the lower crustal flow model^18^. Argand^13^ proposed that the low-angle underthrusting of the Indian slab doubled the continental crustal thickness, which elevated the overriding plate above it. Others have also argued that most or all of the Himalaya-Tibet region has been underplated by Indian continental lithosphere using geological arguments^6^, or geophysical constraints^19–22^. However, other observational studies imply that the underthrusting of the Indian continent below the overriding Eurasian plate only reaches the northern margin of the Himalaya^12,23–25^ or a boundary located somewhere in Southern Tibet^26–31^.

Another point of scientific discussion concerns the possibility of continental subduction at the India-Eurasia convergent zone. Several researchers have proposed that subduction of the positively-buoyant Indian continent is not feasible unless there is significant eclogitization during subduction that increases the density of the continental crust^33–35^. Some modelling works^36,37^ also considered that subduction of the entire crust of the Indian continent is infeasible and proposed that the upper crust has been scrapped off by the overriding plate during continental subduction leaving only the lower Indian crust and lithospheric mantle to subduct into the ambient mantle. However, the above studies did not consider the existence of laterally connected subduction zones, namely the Sunda subduction zone and Makran subduction zone, which also contribute to Indian continental subduction and northward motion, and East Asian deformation^38–41^.

Therefore, geodynamic modelling is required to investigate if Indian continental subduction and flat slab formation are geodynamically feasible (i.e. dynamically feasible on Earth in terms of driving and resistive forces in the context of plate tectonics, subduction and mantle flow), when considering the laterally connected subduction zones (Sunda and Makran subduction zones), if and how the flat slab affects the topography of the Himalaya-Tibet region, and to provide quantitative insights on its horizontal extent. Earlier studies focusing on the subduction process of oceanic lithosphere have shown that subduction of buoyant features on the oceanic lithosphere (e.g. aseismic ridges, seamount chains, oceanic plateaus) can produce flat slab subduction below the base of the overriding plate^42–45^. Such a process can increase the surface topography of the overriding plate directly above the flat slab segment because of the higher average buoyancy of the oceanic lithosphere segment carrying the buoyant feature. Since the Indian continent is positively buoyant with a considerable size and seismic images also show underthrusting of the Indian slab segment at a shallow depth^46^, it is worthwhile to investigate whether subduction of the positively buoyant Indian continent contributes to the high surface topography of the Himalaya and Tibet. Our experimental models build on the upper mantle models of Bose et al.^38^, which demonstrated that lateral subduction zones (Sunda and Makran) are required for northward Indian continental motion and Indian indentation into the Eurasian plate, but which showed relatively limited continental subduction and no continental flat slab subduction. Our models will test the role of deep mantle subduction in driving continental subduction and the formation of a continental flat slab.

In this study, we present three 4-dimensional (3D space + time) buoyancy-driven (fully-dynamic) analogue experiments to investigate whether deep mantle subduction enhances continental subduction of the buoyant Indian lithosphere, if continental subduction can result in a flat slab directly below the base of the overriding plate lithosphere, and, if so, whether the flat slab contributes to the high surface topography above it. Since the amount of Indian continental subduction can be affected by the available slab pull and the resistance of the ambient mantle, we vary the mantle depth and lower–upper mantle viscosity ratio (*η*_*LM*_*/η*_*UM*_) between the experiments: Exp. 1 only has an upper mantle reservoir with an infinitely high *η*_*LM*_*/η*_*UM*_ at the 660-km discontinuity, Exp. 2 has a deep mantle reservoir with no viscosity step at the 660-km discontinuity (*η*_*LM*_*/η*_*UM*_ = 1), and Exp. 3 has a deep mantle reservoir with an intermediate *η*_*LM*_*/η*_*UM*_ (~ 8.6).

## Methodology

**Figure 2:**
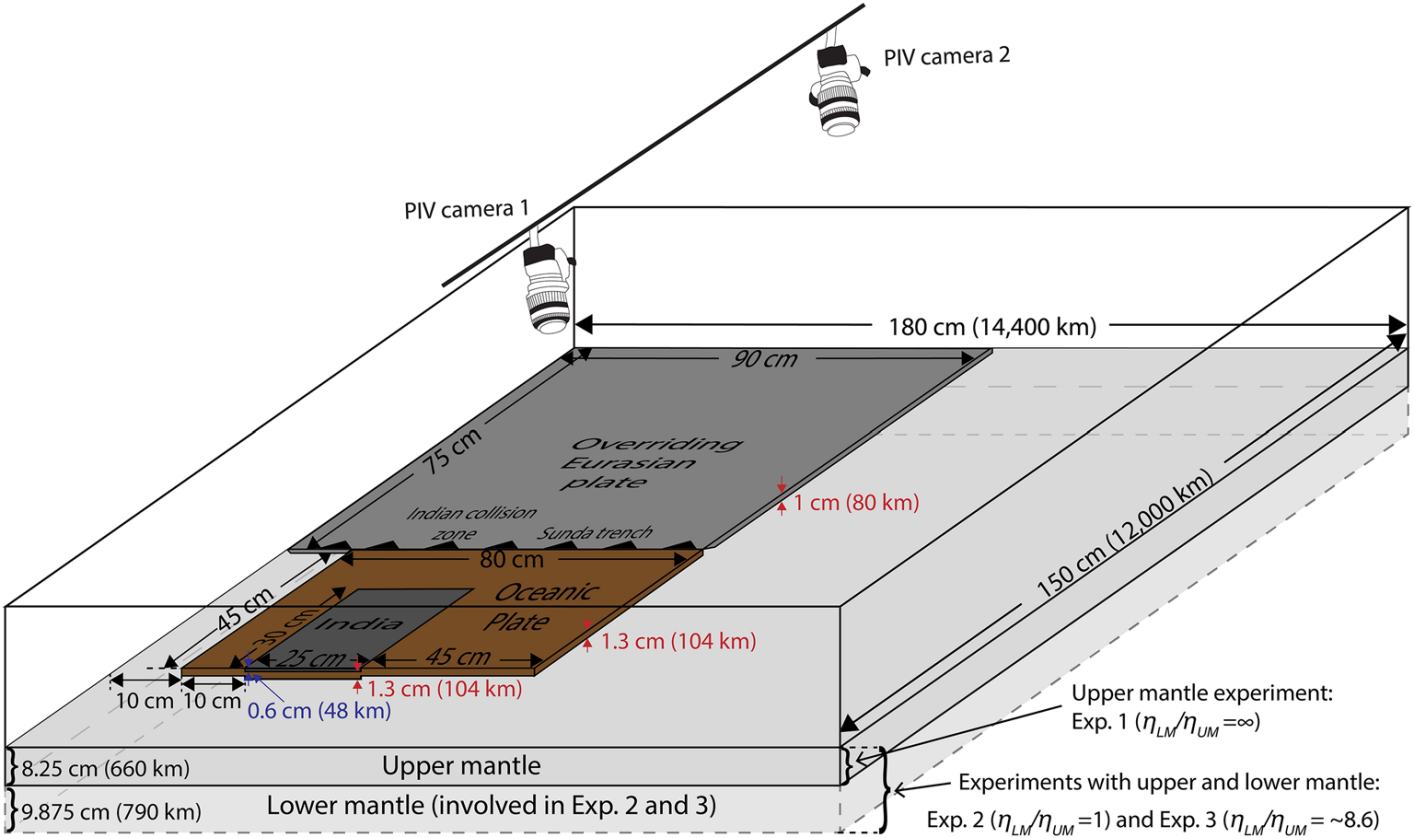
Schematic model setup. Experiment 1 involves only upper mantle material with the rigid bottom of the tank simulating an infinite viscosity step at the 660-km discontinuity. Experiment 2 involves a homogeneous mantle layer with a thickness that represents 1450 km in nature and excludes a viscosity step at the 660-km discontinuity (*η*_*LM*_*/η*_*UM*_ = 1). Experiment 3 incorporates an upper mantle layer with a thickness of 8.25 cm (660 km) and lower mantle layer with a thickness of 9.875 cm (790 km), with a viscosity step at the 660-km discontinuity (*η*_*LM*_*/η*_*UM*_ =  ~ 8.6). Two cameras using a stereo-photogrammetry technique are used to compute the overriding Eurasian plate topography from the top.

We present three buoyancy-driven analogue models to simulate the India-Eurasia-Sunda collision-subduction zone in a big tank with a length of 180 cm and a width of 150 cm, which is filled with different amounts of glucose syrup, either homogeneous or layered, to represent the sub-lithospheric mantle reservoir (Fig. 2). Our models build on the experimental design presented in Bose et al.^38^, who presented buoyancy-driven, upper mantle, India-Eurasia collision-subduction experiments that either included or excluded lateral subduction zones. We improve on the experimental set-up of their most successful experiment (which includes the lateral Sunda and Makran subduction zones) by presenting experiments that include a lower mantle reservoir below the upper mantle.

A single layer of glucose syrup (density *ρ*_*UM*_ = 1408 kg/m^3^ and viscosity *η*_*UM*_ =  ~ 40 Pa s) with a thickness of 8.25 cm (scaling to 660 km in nature) is implemented in Exp. 1 to model the upper mantle reservoir, such that the rigid bottom boundary of the tank simulates the 660 km discontinuity with an infinitely high *η*_*LM*_*/η*_*UM*_. A thicker (18.125 cm, scaling to 1450 km in nature) glucose syrup reservoir is implemented in both Exps. 2 and 3. In Exp. 2, the glucose syrup is homogeneous with a density of 1408 kg/m^3^ and a viscosity of ~ 40 Pa s to model a mantle reservoir with *η*_*LM*_*/η*_*UM*_ = 1. In Exp. 3, the syrup reservoir is stratified with a density of 1396 kg/m^3^, viscosity of ~ 28 Pa s and thickness of 8.25 cm for the upper layer, representing the upper mantle, and a density of 1421 kg/m^3^, viscosity of ~ 240 Pa s and thickness of 9.875 cm for the lower layer, representing the lower mantle, giving a *η*_*LM*_*/η*_*UM*_ of ~ 8.6. The value of *η*_*LM*_*/η*_*UM*_ in Exp. 3 is slightly lower than the lower bound of the estimated ratio in nature (10–100)^47–49^. However, the resulting slightly lower resistance to subduction at the 660-km discontinuity is partly compensated for by the moderate lower–upper mantle density contrast (Δ*ρ*_*LM-UM*_ = 25 kg/m^3^). We note that to dynamically scale our analogue experiments of subduction and collision, we choose to scale the density contrasts between the different layers and plates in our experiments (see below). When choosing this scaling approach, the absolute values of the densities are not important^50^. In our three experiments, these density contrasts are all the same, while the densities are slightly different. We used slightly lower densities for our Exp. 3 compared to Exps. 1 and 2, because we had to reduce the viscosity of our sub-lithospheric upper mantle reservoir (syrup) in Exp. 3 by mixing it with water^51^. This was required to have the correct viscosity ratio between sub-lithospheric upper mantle and lower mantle.

For all three experiments, the overriding Eurasian plate and the oceanic lithosphere both have linear-viscous properties and are made of a mixture of high-viscosity, linear-viscous silicone and iron powder. The Indian continental crust consists of a mixture of 50 wt% of high-viscosity, linear viscous silicone, 39.62 wt% of high-viscosity, pink silicone rubber, and 10.38 wt% of low-viscosity, linear viscous silicone. The sizes of the plates are illustrated in Fig. 2 and a length scaling of 1 cm representing 80 km in nature is applied in this study. A wedge shape with an angle of ~ 30° is made at the leading edge of the Indian continent to simulate the passive margin. The overriding plate has a density of 1408 kg/m^3^ for Exps. 1–2 and 1396 kg/m^3^ for Exp. 3, and is neutrally buoyant relative to the upper mantle material (*ρ*_*UM*_ = 1408 kg/m^3^ for Exps. 1–2 and 1396 kg/m^3^ for Exp. 3). The oceanic lithosphere (*ρ*_*OL*_ = 1508 kg/m^3^ for Exps. 1–2 and 1496 kg/m^3^ for Exp. 3) has a density contrast of 100 kg/m^3^ relative to the upper mantle material, which is slightly higher than that estimated in nature (80 kg/m^3^)^52^ to compensate for the surface tension in the experiments. The Indian continental lithosphere consists of a 0.6 cm thick Indian crust (*ρ*_*InCrust*_ = 1038 kg/m^3^ for Exps. 1–2 and 1026 kg/m^3^ for Exp. 3) and an underlying 1.3 cm thick lithospheric mantle (*ρ*_*LM*_ = 1508 kg/m^3^ for Exps. 1–2 and 1496 kg/m^3^ for Exp. 3), resulting in an average density of ~ 1360 kg/m^3^ for Exps. 1–2 and 1348 kg/m^3^ for Exp. 3, which is positively buoyant relative to the ambient upper mantle material (density contrast of ~ 48 kg/m^3^). A film of lubricant (made of 10wt% petrolatum and 90wt% paraffin oil) with a thickness of ~ 0.5–1.0 mm is applied to the top of the subducting plate, in order to model a weak coupling at the subduction zone plate boundary interface, representing the hydrated uppermost crust and sediments at the subduction interface^53^.

Our models are dynamically scaled to nature according to the Stokes’s settling law following earlier studies^53–55^. The upper mantle in Exp. 1 and the homogeneous upper and lower mantle in Exp. 2 have a viscosity of 40 Pa s, scaling to 6.73 × 10^19^ Pa s in nature. Exp. 3 has a viscosity of ~ 28 Pa s (4.7 × 10^19^ Pa s in nature) in the upper mantle and ~ 240 Pa s (4 × 10^20^ in nature) in the lower mantle. The scaled viscosity values are all within the estimated range of 10^19^—10^21^ Pa s for nature^56,57^. In addition, the overriding Eurasian plate has a viscosity of ~ 2.8 × 10^4^ Pa s (4.7 × 10^22^ Pa s in nature), the subducting oceanic lithosphere has a viscosity of 3.0 × 10^4^ Pa s (5 × 10^22^ Pa s in nature), and the Indian crust has a viscosity of 2.5 × 10^4^ Pa s (4.2 × 10^22^ Pa s in nature). The scaling of time follows from the scaling of viscosity^50^, and in our experiments 1 s represents 8300 years in nature. We calculate the Reynolds number (*Re*) for our experiments following earlier studies^53^ and the maximum values of *Re* are ~ 1.1 × 10^–3^, 6.9 × 10^–3^ and 3.0 × 10^–3^ for Exps. 1–3, respectively. For all experiments, *Re* is much smaller than 1, which means our experiments have dynamic similarity with nature, reproducing a laminar flow regime with upstream–downstream symmetry^58^.

We use density contrasts between the plates and the mantle material to scale our models to nature. Accordingly, we need to apply a topographic correction factor (*C*_*Topo*_ = *ρ*_*m_UM*_/*ρ*_*n_UM*_, where *ρ*_*m_UM*_ and *ρ*_*n_UM*_ represent the density of the upper mantle reservoir in the model and nature, respectively) to scale the topography in our models to that in nature, following Schellart & Strak^50^. Application of this method to our experiments indicates that 1 mm of topography in our models represents ~ 3.47 km in nature.

The experiments are initiated by pouring ~ 30 ml of upper mantle material on top of the first ~ 3 cm of the leading side of the subducting oceanic lithosphere, that forms an initial dip angle of ~ 30°. We calculate the topography of the overriding Eurasian plate with a stereo-photogrammetry technique with a subset size of 25 pixels and a step size of 6 pixels, following the approach as discussed in Chen et al.^59^. We record the slab geometry at the end of the experiments by taking photographs from the top after the overriding plate is manually pulled away to be able to view the geometry of the subducted slab from a top view perspective. The overriding plate was pulled away quickly to avoid the slab being deformed in a viscous manner, because at such high strain rates the response of the silicone mixture is mostly elastic^60^.

## Results

**Figure 3:**
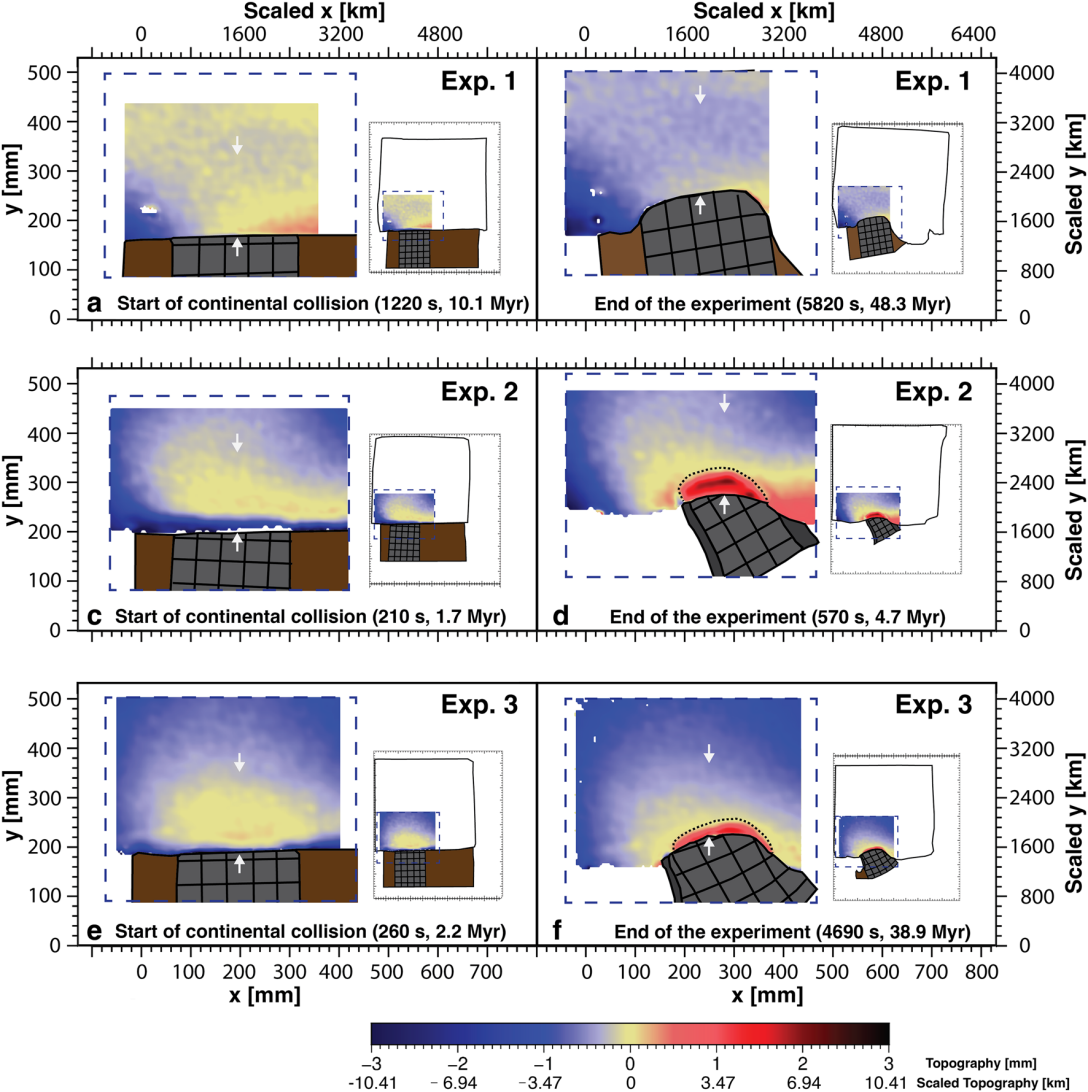
Topographic maps of a sub-area of the overriding plate located at and near the India-Eurasia collisional plate boundary. The location of the sub-area is shown in the inset in the right of each panel. The white arrows represent the location of the topographic profiles in Fig. 5. The dotted lines in panels **d** and **f** indicate the down-dip limit of the continental flat slab.

**Figure 4:**
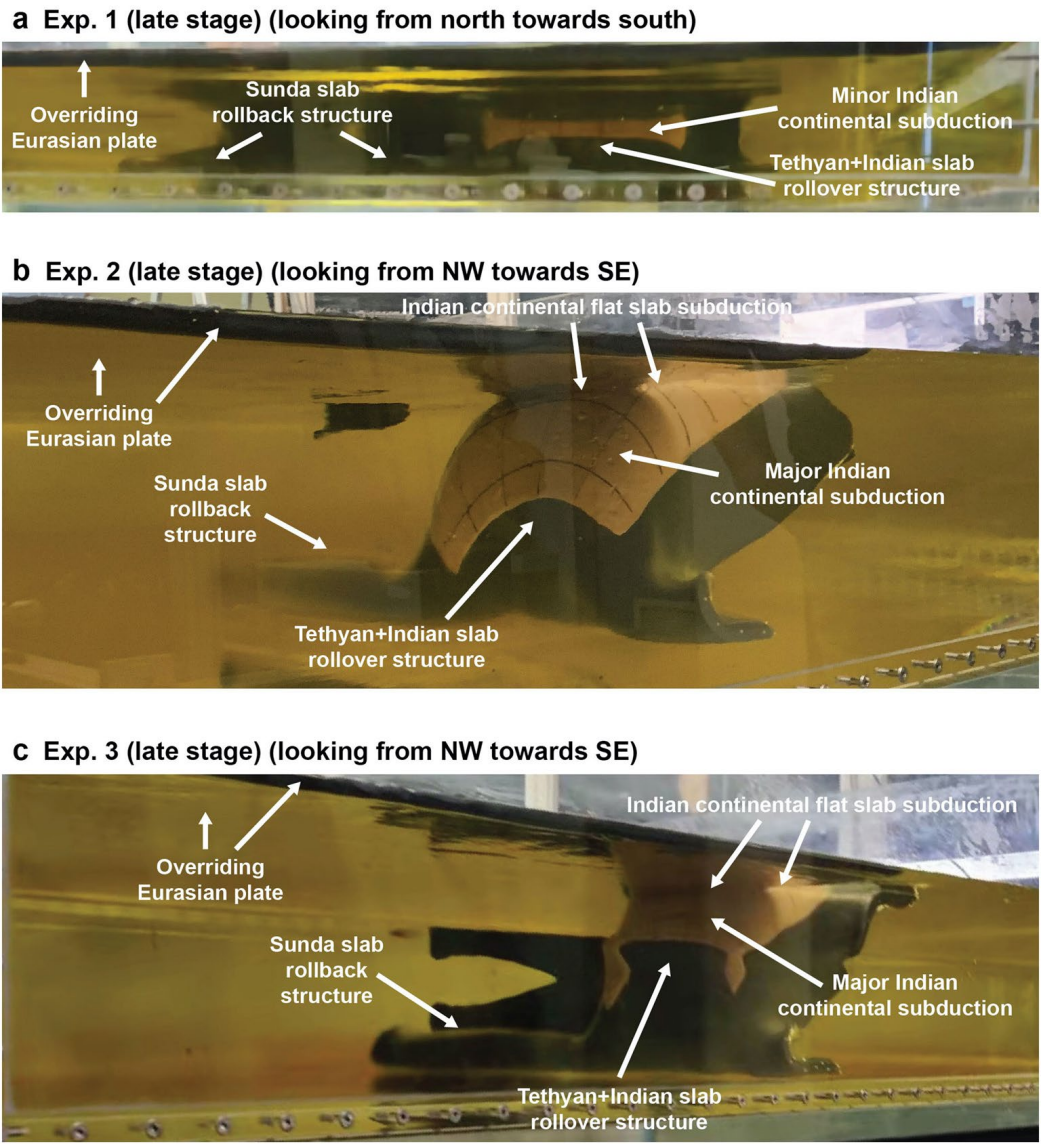
Photographs of the three subduction-collision-continental subduction experiments in a late stage of the experimental evolution for (**a**) Exp. 1 (upper mantle experiment, (**b**) Exp. 2 (deep homogeneous mantle experiment), and (**c**) Exp. 3 (layered mantle experiment). The photographs show the mantle reservoir, the slab structure (oceanic slab is dark grey-black, Indian continental crust is light pink with thin black passive grid lines) and the bottom of the overriding plate. All experiments show a slab rollback and slab draping structure for the Sunda slab and a slab rollover structure for the Tethyan + Indian slab. Exps. 2 and 3 show Indian continental flat slab subduction. Photographs in (**a**) and (**c**) by K. Xue. Photograph in (**b**) by W.P. Schellart.

Our experiments all start with rapid subduction of the leading oceanic lithosphere and the associated increase of the subducting plate velocity, trench velocity, convergence velocity and subduction velocity, and overriding plate extension. At the Sunda subduction zone, the trench motion is dominated by trench retreat from the beginning to the end of the experiments (Fig. 3). At the India-Eurasia collision zone, the motion of the plate boundary changes from retreat to advance and the convergence rate drops after reaching a maximum of ~ 14 cm/yr when the slab tip reaches the 660-km discontinuity for Exp. 1. In Exps. 2 and 3 the change from retreat to advance and the decrease in convergence rate after maxima of ~ 86 and ~ 26 cm/yr, respectively, occur when the Indian continent arrives at the plate boundary. In all experiments, the trench retreat at the Sunda subduction zone corresponds with a slab draping geometry, while the plate boundary advance at the India-Eurasia collision zone corresponds with a slab roll-over geometry in the experiments (Fig. 4). All experiments show continental subduction of the Indian continental lithosphere, but in Exp. 1 only minor continental subduction takes place (~ 1 cm scaling to ~ 80 km), while in Exps. 2 and 3 major continental subduction occurs (~ 12 cm scaling to ~ 960 km for Exp. 2 and ~ 7 cm scaling to ~ 560 km for Exp. 3) (Table 1, Fig. 4).

**Table 1:** Measurements of the elevated topography (bulge) and flat slab length near the collisional plate boundary formed during long-term Indian indentation, and the amount of continental subduction.

Experiment number	Bulge			Subducted amount of Indian continent [cm] ([km])	Flat slab length [cm] ([km])
	Trench-normal length [cm] ([km])	Maximum height [mm] ([km])	Average height [mm] ([km])		
Exp. 1				~ 1 (80)	0
Exp. 2	~ 6.0 (480)	~ 2.1 (7.3)	1.2 (4.2)	~ 12 (960)	~ 5.3 (424)
Exp. 3	~ 3.5 (280)	~ 1.1 (3.8)	0.5 (1.7)	~ 7 (560)	~ 4 (320)

The length of the bulge and the flat slab are all measured along the mid axis of the Indian continent in the trench-normal direction.

### Topography

**Figure 5:**
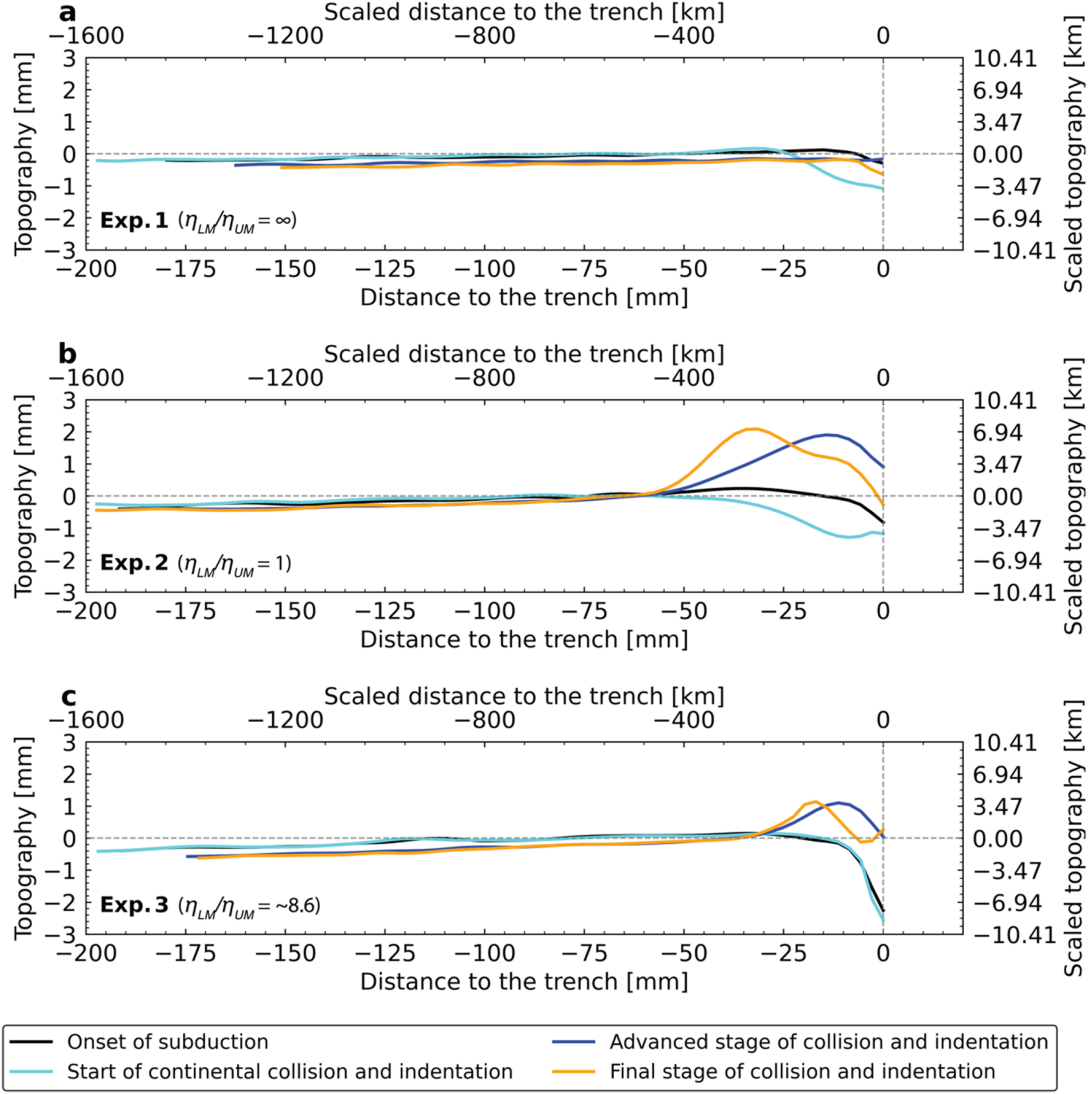
Diagrams showing the topographic profiles of the overriding Eurasian plate north of India at four times for Exps. 1–3. (**a**–**c**) Start of subduction (start of the experiment) (black lines), start of continental collision and indentation (aqua lines), advanced stage of collision and indentation (blue lines), and final stage of the collision and indentation (end of the experiment) (orange lines) for the three experiments. The position of the topographic profiles is indicated by white arrows in Fig. 3.

Before the onset of India-Eurasia collision, the frontal part of the overriding Eurasian plate is dragged down for all experiments (Fig. 3a,c,e, black and aqua lines in Fig. 5). This area is dragged down by a maximum of ~ 0.8, 0.4 and 0.3 mm (2.8, 1.4, 1 km) for Exps. 1–3, respectively, from the beginning of the experiment (black lines in Fig. 5) to the onset of India-Eurasia collision (aqua lines in Fig. 5). The trench-normal extent of this area varies with time and per experiment, but generally ranges between ~ 10 and ~ 40 mm (scaling to 80–320 km).

From the onset of continent–continent collision and during the long-term Indian indentation, the frontal area of the overriding Eurasian plate is elevated in all experiments (Fig. 3b,d,f, Fig. 5), with a trench-normal extent of ~ 20, 60 and 35 mm (160, 480 and 280 km) for Exps. 1–3, respectively. The least amount of uplift is observed for Exp. 1, which shows a maximum of ~ 0.5 mm (1.7 km) of uplift during the collision stage (i.e. maximum elevation difference between orange line and aqua line in Fig. 5a), and the elevation at the end of the experimental run is below the one observed at the start of the experiment by ~ 0.2 mm (compare orange and black lines). In contrast, Exps. 2 and 3 show significant overall uplift in the frontal area of the overriding plate (with respect to the zero elevation line), forming a bulge with a maximum height of ~ 2.1 mm (scaling to 7.3 km, orange line Fig. 5b) and 1.1 mm (scaling to 3.8 km, orange line Fig. 5c), and an average height of ~ 1.2 mm (scaling to 4.2 km) and 0.5 mm (scaling to 1.7 km), respectively. During the entire Indian indentation phase, the topographic bulge in Exps. 2 and 3 shows an overall movement towards the overriding plate, while the height of the bulge does not show any obvious change (compare blue and orange lines in Figs. 5b,c).

### Flat slab

**Figure 6:**
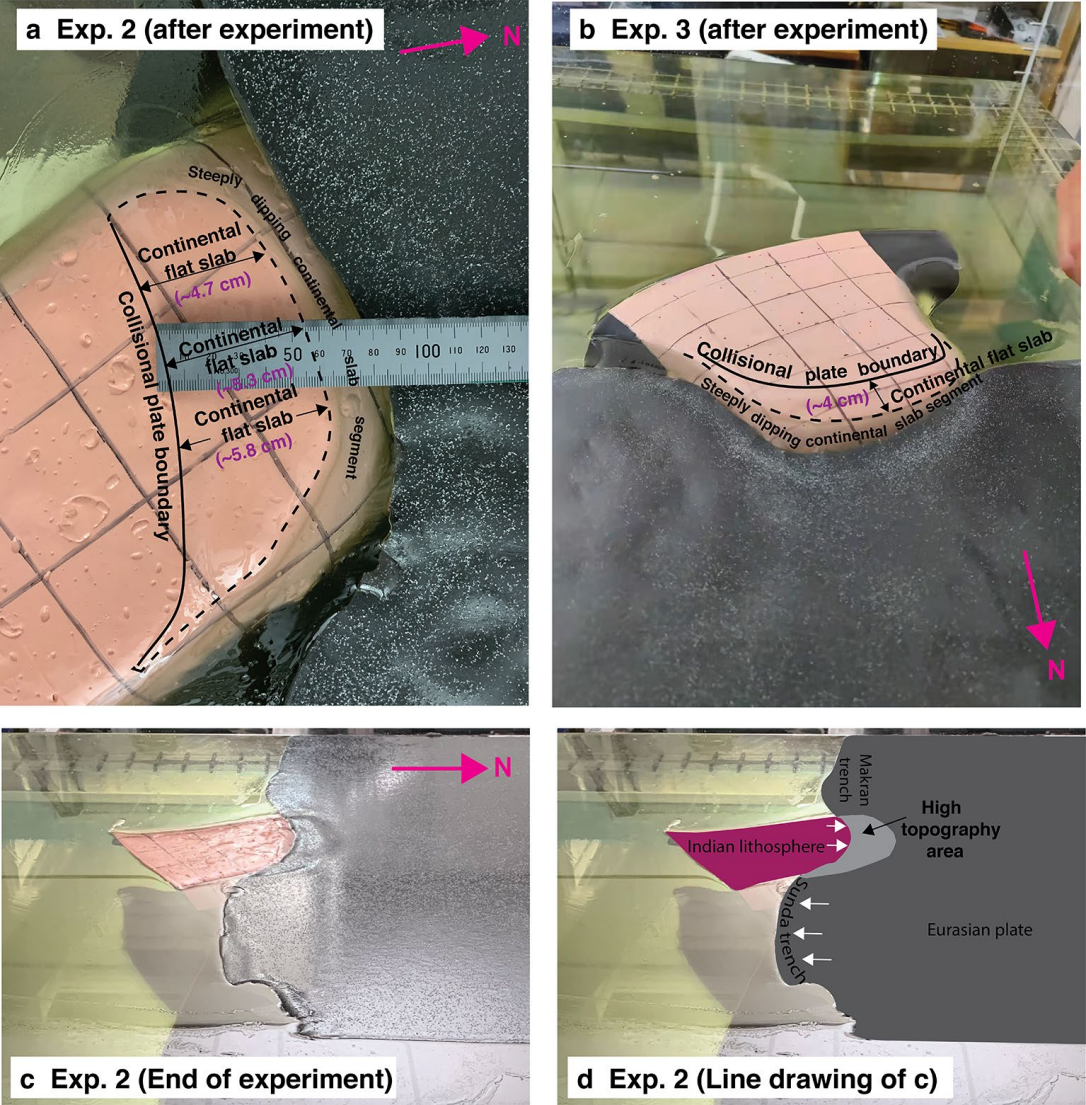
Photographs of the continental flat slab for (**a**) Exp. 2, and (**b**) Exp. 3 which were taken after the overriding plate was manually pulled away to be able to view the subducted slab from above. Photograph in (**a**) shows a top-view perspective, while photograph in (**b**) shows a 3D perspective view. The solid line indicates the collisional plate boundary, while the dashed line delineates the downdip limit of the flat slab, which has a (sub)horizontal orientation and lies directly below the overriding plate, but not below the top surface of the upper mantle reservoir. (**c**) Photograph with a 3D perspective view of Exp. 2 at the end of the experimental run showing the elevated topography in the overriding plate at the India-Eurasia collision zone plate boundary. (**d**) Line drawing of photograph in (**c**). The white arrows in (**d**) indicate the direction of the plate boundary motion. Photographs in (**a**) and (**c**) by W.P. Schellart. Photograph in (**b**) by K. Xue.

After each experiment, the overriding plate is manually pulled away horizontally along the top surface to be able to view and investigate the geometry of the Indian continental slab. The geometry of the Indian continental slab is also visible and investigated from side view images (Fig. 4). For Exp. 1, only ~ 1 cm (80 km in nature) of the Indian continental lithosphere subducts below the overriding Eurasian plate and it dips steeply into the mantle (Fig. 4a). For Exps. 2 and 3, significantly more of the Indian continental lithosphere is subducted (12 cm, scaling to 960 km for Exp. 2, and 7 cm, scaling to 560 km for Exp. 3) (Table 1) (Fig. 4b,c). From this total length of subducted continental lithosphere, some ~ 44 (Exp. 2) and 57% (Exp. 3) closest to the trench underlies the overriding plate horizontally (Fig. 6), which we refer to as continental flat slab subduction, while the remaining ~ 56% (Exp. 2) and 43% (Exp. 3) of the continental lithosphere dips steeply into the mantle. The trench-normal length of the continental flat slab area amounts to ~ 4.7–5.8 cm (376–464 km in nature) and ~ 2.5–4 ± 0.5 cm (200–320 ± 40 km in nature) for Exps. 2 and 3, respectively. The western side of the flat slab area is somewhat narrower, compared to the eastern side for both experiments (Fig. 6).

## Discussion

### High topography and continental flat slab subduction

**Figure 7:**
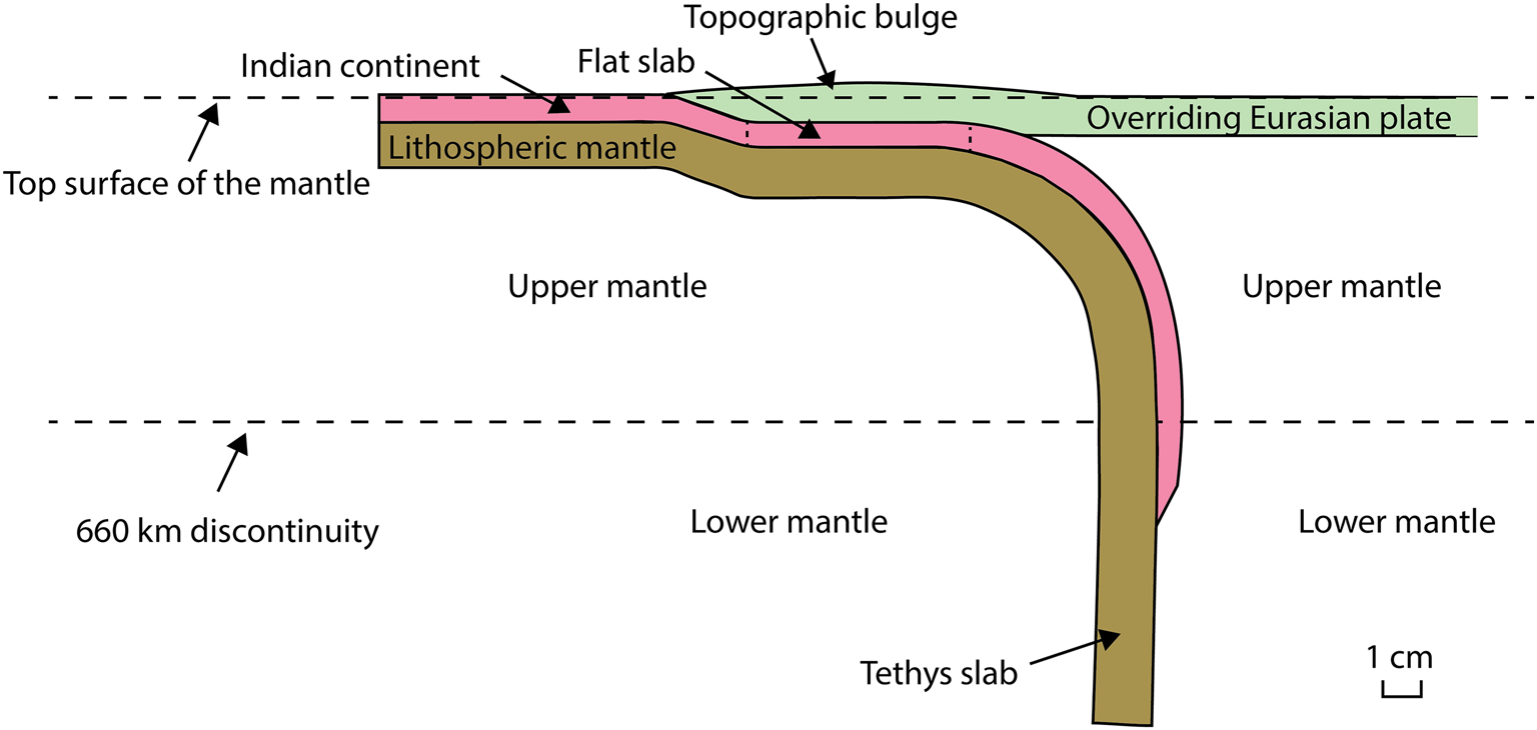
Schematic cross-section illustrating the underthrusting of the experimental Indian continent below the experimental overriding Eurasian plate in our Exps. 2 and 3, forming a topographic bulge above the flat slab segment. The length scale of the bulge and flat slab refers to Exp. 2.

Previous geodynamic models have shown that during trench advance due to oceanic subduction, the topography of the overriding plate near the trench is lower compared to the trailing part of the overriding plate and that this low topography mainly results from the vertical component of the shear force at the subduction interface^61^. Contrastingly, subduction of the buoyant Indian continental lithosphere during collisional plate boundary advance leads to a bulge (Fig. 7) with high topography next to the plate boundary in our Exp. 2 (maximum height scales to ~ 7.3 km and average height scales to ~ 4.2 km) and Exp. 3 (maximum height scales to 3.8 km and average height scales to ~ 1.7 km). An important observation is that the trench-normal extent of the continental flat slab and the topographic bulge are comparable (Table 1). On the other hand, in our Exp. 1 with only 1 cm (80 km) of continental subduction, both a topographic bulge at the surface and a flat slab are not observed. In addition, our models do not simulate crustal thickening due to orogenic wedge growth and the overriding Eurasian plate in our models is neutrally buoyant and so any thickening of this plate will not produce any topography. Therefore, we propose that the high topographic area of the overriding plate bordering the collisional plate boundary in our models results from the underlying continental flat slab. This can be explained by the positive buoyancy of the subducted Indian lithosphere, of which the subduction is driven by the slab pull from the Tethyan oceanic lithosphere in the upper and lower mantle, as well as the slab pull from the lateral Sunda and Makran subduction zones. The positive buoyancy of the Indian continental lithosphere (Indian crust + lithospheric mantle) promotes flat slab formation and provides an upward force to the base of the overlying Eurasian overriding plate, thereby forming a high surface topography.

The trench-normal extent of the flat slab, the trench-normal extent of the bulge, and the height of the bulge are all higher in Exp. 2, compared to Exp. 3 (Table 1). The higher extent of the flat slab in Exp. 2 can be partly explained by the higher slab pull in Exp. 2 that resulted from the lower resistance of subduction at the 660-km discontinuity (low *η*_*LM*_*/η*_*UM*_), compared to Exp. 3. The lower resistance at the 660-km discontinuity and large slab pull force promote faster subduction and cause more Indian continent to be dragged into the ambient mantle in Exp. 2 (~ 12 cm, scaling to 960 km), compared to Exp. 3 (~ 7 cm, scaling to 560 km). Considering that the continental lithosphere is positively buoyant, then more subduction of this lithosphere will promote a longer flat slab segment, and therefore the extent of the flat slab segment is also larger in Exp. 2 (5.3 cm long in the trench-normal direction, scaling to 424 km), compared to Exp. 3 (4 cm long in the trench-normal direction, scaling to 320 km). The longer flat slab segment leads to a bigger bulge in the overriding plate above the flat slab in Exp. 2 (~ 32.5% longer than Exp. 3). Additionally, the much faster subduction in Exp. 2 causes the mantle flow to be stronger, which results in a higher mantle wedge suction force, thereby promoting a longer flat slab segment and thus a larger topographic bulge compared to Exp. 3.

During the long-term Indian indentation and subduction, an obvious change of the bulge height is not observed in Exps. 2 and 3. This can be explained by the fact that the vertical forces applied to the overriding plate above the flat slab, which result from the buoyancy force of the Indian continent, do not vary much during continental subduction. The main change we observe is a northward displacement of the topographic bulge that can be attributed to the northward plate boundary advance.

### Implications for the Himalaya-Tibet collision system

There exists considerable debate and controversy on the ~ northward extent of Indian underthrusting below the Himalaya-Tibet orogen. There are three main groups with different interpretations for the extent of Indian underthrusting/continental flat slab subduction below the overriding Eurasian plate, which are: (1) underthrusting mainly below the Himalaya extending ~ 200–290 km from the MFT (~ 280–290 km from Makovsky et al.^24^; ~ 200 km from Shi et al.^31^; ~ 240 km from Zhao et al.^12^, (2) underthrusting below the Himalaya and part of Southern Tibet extending ~ 260–440 km from the MFT (~ 260–410 km from Klemperer et al.^30^; ~ 330–440 km from Nábelek et al.^29^), and (3) underthrusting below the Himalaya and most/all of Tibet extending ~ 600–1050 km from the MFT (~ 600–900 km from Barazangi & Ni^19^; ~ 600–950 km from Chen et al.^23^; ~ 800 km from Mckenzie et al.^21^; ~ 750–1050 km from Zhou & Murphy^22^). There are also papers that show very wide ranges of underthrusting, such as ~ 350–700 km^46^. The ~ N-S extent of the continental flat slab segment produced in our Exp. 2 is ~ 376–464 km, which is higher than the ~ N-S extent of the Himalaya (~ 200–320 km) and thus covers part of Southern Tibet, in agreement with the above group 2. In our Exp. 3, the ~ N-S extent of the flat slab is ~ 320 km in the centre, while the ~ N-S extent of the Himalaya in the centre is ~ 200 km. Therefore, our Exp. 3 predicts that ~ 120 km of Southern Tibet is also underthrusted, which is comparable with the estimates from group 2.

In our Exp. 2, the slab pull is maximized, because there is no viscosity step at the 660 km discontinuity, producing a greater flat slab extent. However, in nature, the viscosity step is much higher than 1 and its estimated minimum is close to ~ 8.6 (as in our Exp. 3). Thus, the extent of the flat slab, in the absence of other processes that could promote continental subduction and flat slab formation, should be lower than that estimated in our Exp. 2, but more comparable to that observed in our Exp. 3. Considering the above, our values of the maximum flat slab extent from Exp. 2 provide an upper bound as to what is geodynamically viable, even more so considering that the slab in Exp. 2 is continuous from the surface down to the lower mantle. Seismic tomography studies of the Himalayan region, however, imply that the slab is not continuous in many places along the collisional plate boundary^46,62,63^ (Fig. 1a,b), likely due to slab detachment and break-off processes in the upper mantle. Such detachment and break-off will also lower the amount of slab pull^64,65^.

The importance of the conclusion of an upper bound for the extent of the continental flat slab as provided by Exp. 2 is that this upper bound is significantly lower compared to the values proposed by the above group 3. Furthermore, the results of our preferred experiment indicate that the continental flat slab can extend some 320 km northward from the MFT, suggesting that Indian underthrusting and continental flat slab subduction occur mainly below the Himalaya and part of Southern Tibet, in accordance with values reported in group 2 above.

In our experiments, the overriding plate experiences horizontal shortening due to northward indentation of India, with maximum shortening values of the order 80, 20 and 40% for Exps. 1, 2 and 3, respectively. The horizontal shortening and vertical thickening do not result in any topography, because our overriding plate is neutrally buoyant with respect to the sub-lithospheric upper mantle. Due to this neutral buoyancy, our experiments allow to directly quantify the contribution of the underthrusting of the Indian continental lithosphere to the uplift of the Himalaya-Tibet region. Our Exps. 2 and 3 present a bulge with an average elevation of ~ 4.2 and 1.7 km, which represent ~ 84% and 34% of the average elevation of the Himalaya (~ 5 km), respectively. Observational studies indicate a much higher crustal thickness reaching > 80 km beneath the Himalaya-Tibet orogen^66,67^ compared to the average thickness of the continental crust in nature (~ 35 km^68,69^), which suggests a considerable contribution of the crustal thickening to the high topography of the Himalaya-Tibet region. Since our Exp. 3 has a viscosity step at the 660-km discontinuity that is the closest to nature, we suggest that the buoyant flat slab contributes as much as ~ 34% to the high topography of the Himalaya-Southern Tibet region, while other factors (e.g. crustal thickening) would contribute to the remaining part of the high topography. This contribution to the high topography in Exp. 3 is limited to a region within ~ 320 km and located north of the collisional plate boundary. As such, it does not reproduce, nor does it provide, an explanation for the high elevation of the Tibetan plateau northward of this ~ 320 km boundary. Nevertheless, our models do indicate that underthrusting and continental flat slab subduction below the entire Tibetan Plateau are dynamically not viable, and thus our models do not support the conceptual models from e.g. Argand^13^ and Powell & Conaghan^6^ in which the high topography of the Tibetan Plateau results entirely from this underthrusting and continental flat slab subduction.

### Comparison with previous models of flat slab subduction

To the best of our knowledge, these are the first reported buoyancy-driven geodynamic models of continental flat slab subduction. Flat slab subduction (with oceanic lithosphere) has been reported before in various modelling studies, both analogue and numerical, but these generally involved kinematic boundary conditions^43–45,70–77^. Only a few examples exist of buoyancy-driven modelling studies reporting (oceanic) flat slab subduction^78,79^. Some geodynamic modelling studies of flat slab formation at normal (oceanic) subduction zones have shown that the subduction of positively buoyant features (e.g. aseismic ridge, seamount chain, oceanic plateau) can lead to the formation of a flat slab^43–45,71^. Models from Espurt et al.^71^ and Martinod et al.^45^ both showed a flat slab and a high topography above the flat slab segment. However, the high topography (e.g. scaling to ~ 20 km in Espurt et al.^71^) in their models is much higher compared to that in our models and in nature. This high topography could be caused by the high velocities, which resulted from external driving forces in their models. Our Exps. 2–3 produce realistic topographies (maximum elevation reaching ~ 7.3 and 3.8 km, average elevation of ~ 4.2 and 1.7 km for Exps. 2 and 3, respectively) without an external driving force and this can be explained by the buoyancy-driven approach used in our models, that produce relatively lower velocities and forces during subduction and thus relatively lower topography. A recent buoyancy-driven analogue modelling study on the subduction of an aseismic ridge^80^ did not produce a flat slab, but a lower slab dip angle at the ridge location was observed in one of their models, in which the ridge is the thickest (~ 20 km) and widest (200–400 km). The difference in outcome between their experiments (no flat slab) and our Exps. 2 and 3 (with flat slab) can be ascribed to various factors, including a much bigger size of the buoyant feature (Indian crust, scaling to 48 km thick and 2000 km wide), a much higher slab pull force because of the much deeper subduction of the slab into the lower mantle, and the existence of a much larger lateral subduction segment (Sunda) in our experiments.

Earlier geodynamic models of the India-Eurasia collision have been presented, both analogue^81,82^ and numerical^83^, but these have not reproduced continental flat slab subduction. Replumaz et al.^81^ and Pitard et al.^82^ presented India-Eurasia collision-continental subduction experiments that involved an external velocity boundary condition (piston) and which excluded lateral subduction zones. The absence of continental flat slab subduction in these experiments might be due to the relatively narrow width of the convergent plate boundary, scaling to only 1200 km in both studies (much narrower than in the current study, where it scales to 6400 km). Such a narrow width would cause mantle wedge suction forces to be relatively small^78,84^, thereby preventing a flat slab to form. Pusok & Kaus^83^ presented models with and without an external driving force for Indian continental subduction. A high topography in front of the collisional boundary was only observed in their models with an external driving force, which was due to the crustal thickening, but not in their fully-dynamic model, which was driven only by the negative buoyancy of the oceanic slab. This could result from the insufficient subduction of Indian continent in their fully-dynamic models, thereby inhibiting a flat slab to form. This insufficient amount of Indian continental subduction could be explained by two main differences between their and our study. The first is the relatively lower negative buoyancy force in front of the Indian continent, which was caused by both the smaller mantle thickness implemented in their model (1000 km in their work vs. 1450 km in our models) and shorter Tethyan subducted segment before Indian continental subduction (< 1000 km in their work vs. ~ 1200 km in our models). The other difference is that their model implemented a narrower (~ 1500 km measured along the trench) Sunda subduction zone compared to that in nature and in our models (~ 3600 km), which diminished the role of the lateral oceanic subduction zone in driving continental subduction.

## Conclusion

Our self-consistent, buoyancy-driven geodynamic experiments have demonstrated that large-scale underthrusting/continental flat slab subduction is physically viable in a continental subduction-indentation-subduction setting such as found at the India-Eurasia-Sunda convergent zone. Furthermore, our models provide new insights and understanding of the slab geometry at the India-Eurasia continental subduction zone and the high topography of the Himalaya-Tibet region. Our models demonstrate that a lower *η*_*LM*_*/η*_*UM*_ promotes the subduction of positively buoyant continental lithosphere, while an increased amount of positively buoyant continental lithospheric subduction facilitates the formation of a flat slab. The positively buoyant flat slab that forms below the overriding plate provides an upward force to the overlying plate, forming a high surface topography at the leading edge of the overriding plate overlying the flat slab. Our models suggest that part of the high topography of the Himalaya and southernmost Tibet, on average possibly some 1.5–2 km, is related to the underthrusting of the positively buoyant Indian continental lithosphere directly beneath the base of the overriding Eurasian plate. Our self-consistent models provide a geodynamic constraint on the upper limit of the northern extent of Indian continental flat slab subduction, which is of the order 320 + /– 40 km from the Main Frontal Thrust.

## Data Availability

The datasets generated during the current study are available in FigShare repository (Xue, Kai; Schellart, Wouter Pieter; Strak, Vincent (2022). Geodynamic models of Indian continental flat slab subduction with implications for the topography of Himalaya-Tibet region. figshare. Dataset. https://doi.org/10.6084/m9.figshare.19701217.v2). All data generated or analysed during this study are included in this published article.

## References

[1] Fielding E, Isacks B, Barazangi M, Duncan C (1994). How flat is Tibet?. Geology.

[2] Allegre CJ, Courtillot V, Tapponnier P (1984). Structure and evolution of the Himalaya-Tibet orogenic belt. Nature.

[3] Bouilhol P, Jagoutz O, Hanchar JM, Dudas FO (2013). Dating the India-Eurasia collision through arc magmatic records. Earth Planet. Sci. Lett..

[4] Hu X (2016). The timing of India-Asia collision onset—Facts, theories, controversies. Earth Sci. Rev..

[5] Huang W (2017). Remagnetization of the Paleogene Tibetan Himalayan carbonate rocks in the Gamba area: Implications for reconstructing the lower plate in the India-Asia collision. J. Geophys. Res. Solid Earth.

[6] Powell CMA, Conaghan PJ (1973). Plate tectonics and the Himalayas. Earth Planet. Sci. Lett..

[7] Replumaz A, Kárason H, van der Hilst RD, Besse J, Tapponnier P (2004). 4-D evolution of SE Asia’s mantle from geological reconstructions and seismic tomography. Earth Planet. Sci. Lett..

[8] Searle MP (1987). The closing of Tethys and the tectonics of the Himalaya. Geol. Soc. Am. Bull..

[9] Sengör AMC (1984). The cimmeride orogenic system and the tectonics of Eurasia. Geol. Soc. Am. Spec. Pap..

[10] DeCelles PG, Robinson DM, Zandt G (2002). Implications of shortening in the Himalayan fold-thrust belt for uplift of the Tibetan Plateau. Tectonics.

[11] Guillot S (2003). Reconstructing the total shortening history of the NW Himalaya. Geochem. Geophys. Geosyst..

[12] Zhao W (1993). Deep seismic reflection evidence for continental underthrusting beneath southern Tibet. Nature.

[13] Argand E (1924). La tectonique de l’Asie. Proc. 13th Int. Geol. Cong..

[14] Davy P, Cobbold PR (1988). Indentation tectonics in nature and experiment. 1. Experiments scaled for gravity. Bull. Geol. Inst. Uppsala.

[15] Tapponnier P (2001). Oblique stepwise rise and growth of the Tibet plateau. Science.

[16] Houseman G, England P (1993). Crustal thickening versus lateral expulsion in the Indian-Asian continental collision. J. Geophys. Res. Solid Earth.

[17] England P, Houseman G (1986). Finite strain calculations of continental deformation: 2. Comparison With the India-Asia Collision zone. J. Geophys. Res. Solid Earth.

[18] Royden LH (1997). Surface deformation and lower crustal flow in eastern Tibet. Science.

[19] Barazangi M, Ni J (1982). Velocities and propagation characteristics of Pn and Sn beneath the Himalayan arc and Tibetan plateau: Possible evidence for underthrusting of Indian continental lithosphere beneath Tibet. Geology.

[20] Craig TJ, Copley A, Jackson J (2012). Thermal and tectonic consequences of India underthrusting Tibet. Earth Planet. Sci. Lett..

[21] McKenzie D, Jackson J, Priestley K (2019). Continental collisions and the origin of subcrustal continental earthquakes. Can. J. Earth Sci..

[22] Zhou H, Murphy MA (2005). Tomographic evidence for wholesale underthrusting of India beneath the entire Tibetan plateau. J. Asian Earth Sci..

[23] Chen M (2017). Lithospheric foundering and underthrusting imaged beneath Tibet. Nat. Commun..

[24] Makovsky Y (1996). Structural elements of the southern Tethyan Himalaya rust from wide-angle seismic data. Tectonics.

[25] Schulte-Pelkum V (2005). Imaging the Indian subcontinent beneath the Himalaya. Nature.

[26] Xu Q, Zhao J, Yuan X, Liu H, Pei S (2015). Mapping crustal structure beneath southern Tibet: Seismic evidence for continental crustal underthrusting. Gondwana Res..

[27] Xu Q, Zhao J, Yuan X, Liu H, Pei S (2017). Detailed configuration of the underthrusting Indian lithosphere beneath Western Tibet revealed by receiver function images. J. Geophys. Res. Solid Earth.

[28] Kind R (2002). Seismic images of crust and upper mantle beneath Tibet: Evidence for Eurasian plate subduction. Science.

[29] Nábelek J (2009). Underplating in the Himalaya-Tibet collision zone revealed by the Hi-CLIMB experiment. Science.

[30] Klemperer SL (2022). Limited underthrusting of India below Tibet: 3He/4He analysis of thermal springs locates the mantle suture in continental collision. Proc. Natl. Acad. Sci. U.S.A..

[31] Shi D (2015). Receiver function imaging of crustal suture, steep subduction, and mantle wedge in the eastern India-Tibet continental collision zone. Earth Planet. Sci. Lett..

[32] Amaru, M. Global travel time tomography with 3-D reference models. Geologica Ultraiectina, Vol. 274 (2007).

[33] Hetényi G (2007). Density distribution of the India plate beneath the Tibetan plateau: Geophysical and petrological constraints on the kinetics of lower-crustal eclogitization. Earth Planet. Sci. Lett..

[34] Ingalls M, Rowley DB, Currie B, Colman AS (2016). Large-scale subduction of continental crust implied by India-Asia mass-balance calculation. Nat. Geosci..

[35] Austrheim H (1991). Eclogite formation and dynamics of crustal roots under continental collision zones. Terra Nova.

[36] Capitanio FA, Morra G, Goes S, Weinberg RF, Moresi L (2010). India-Asia convergence driven by the subduction of the Greater Indian continent. Nat. Geosci..

[37] Toussaint G, Burov E, Avouac JP (2004). Tectonic evolution of a continental collision zone: A thermomechanical numerical model. Tectonics.

[38] Bose S, Schellart WP, Strak V, Duarte JC, Chen Z (2023). Sunda subduction drives ongoing India-Asia convergence. Tectonophysics.

[39] Parsons AJ, Sigloch K, Hosseini K (2021). Australian plate subduction is responsible for northward motion of the India-Asia Collision Zone and ∼1000 km lateral migration of the Indian slab. Geophys. Res. Lett..

[40] Patriat P, Achache J (1984). India-Eurasia collision chronology has implications for crustal shortening and driving mechanism of plates. Nature.

[41] Schellart WP, Chen Z, Strak V, Duarte JC, Rosas FM (2019). Pacific subduction control on Asian continental deformation including Tibetan extension and eastward extrusion tectonics. Nat. Commun..

[42] Antonijevic SK (2015). The role of ridges in the formation and longevity of flat slabs. Nature.

[43] van Hunen J, van Den Berg AP, Vlaar NJ (2002). On the role of subducting oceanic plateaus in the development of shallow flat subduction. Tectonophysics.

[44] Gerya TV, Fossati D, Cantieni C, Seward D (2009). Dynamic effects of aseismic ridge subduction: Numerical modelling. Eur. J. Mineral..

[45] Martinod J (2013). Effect of aseismic ridge subduction on slab geometry and overriding plate deformation: Insights from analogue modeling. Tectonophysics.

[46] Replumaz A, Guillot S, Villaseñor A, Negredo AM (2013). Amount of Asian lithospheric mantle subducted during the India/Asia collision. Gondwana Res..

[47] Hager BH (1984). Subducted slabs and the geoid: Constraints on mantle rheology and flow. J. Geophys. Res..

[48] Kaufmann G, Lambeck K (2000). Mantle dynamics, postglacial rebound and the radial viscosity profile. Phys. Earth Planet. Inter..

[49] Steinberger B, Calderwood AR (2006). Models of large-scale viscous flow in the Earth’s mantle with constraints from mineral physics and surface observations. Geophys. J. Int..

[50] Schellart WP, Strak V (2016). A review of analogue modelling of geodynamic processes: Approaches, scaling, materials and quantification, with an application to subduction experiments. J. Geodyn..

[51] Schellart WP (2011). Rheology and density of glucose syrup and honey: Determining their suitability for usage in analogue and fluid dynamic models of geological processes. J. Struct. Geol..

[52] Cloos M (1993). Lithospheric buoyancy and collisional orogenesis - Subduction of oceanic plateaus, continental margins, island arcs, spreading ridges, and seamounts. Geol. Soc. Am. Bull..

[53] Duarte JC, Schellart WP, Cruden AR (2013). Three-dimensional dynamic laboratory models of subduction with an overriding plate and variable interplate rheology. Geophys. J. Int..

[54] Xue K, Schellart WP, Strak V (2020). Effect of plate length on subduction kinematics and slab geometry: insights From buoyancy-driven analog subduction models. J. Geophys. Res. Solid Earth.

[55] Schmeling H (2008). A benchmark comparison of spontaneous subduction models-Towards a free surface. Phys. Earth Planet. Inter..

[56] Artyushkov EV (1983). Geodynamics.

[57] Ranalli G (1995). Rheology of the Earth.

[58] Schellart WP (2008). Kinematics and flow patterns in deep mantle and upper mantle subduction models: Influence of the mantle depth and slab to mantle viscosity ratio. Geochem. Geophys. Geosyst..

[59] Chen Z, Schellart WP, Duarte JC, Strak V (2017). Topography of the overriding plate during progressive subduction: A dynamic model to explain forearc subsidence. Geophys. Res. Lett..

[60] Weijermars R (1986). Flow behaviour and physical chemistry of bouncing putties and related polymers in view of tectonic laboratory applications. Tectonophysics.

[61] Xue K, Schellart WP, Strak V (2022). Overriding plate deformation and topography during slab rollback and slab rollover: Insights from subduction experiments. Tectonics.

[62] van Der Voo R, Spakman W, Bijwaard H (1999). Tethyan subducted slabs under India. Earth Planet. Sci. Lett..

[63] Li C, van der Hilst RD, Meltzer AS, Engdahl ER (2008). Subduction of the Indian lithosphere beneath the Tibetan Plateau and Burma. Earth Planet. Sci. Lett..

[64] Laik A, Schellart WP, Strak V (2023). Sustained indentation in 2-D models of continental collision involving whole mantle subduction. Geophys. J. Int..

[65] van Hunen J, Allen MB (2011). Continental collision and slab break-off: A comparison of 3-D numerical models with observations. Earth Planet. Sci. Lett..

[66] Koulakov I (2015). Variations of the crustal thickness in Nepal Himalayas based on tomographic inversion of regional earthquake data. Solid Earth.

[67] Laske G, Masters G (2013). Update on CRUST1.0: A 1-degree global model of Earth’s crust. EGU Gen. Assem..

[68] Hacker BR, Kelemen PB, Behn MD (2015). Continental lower crust. Annu. Rev. Earth Planet. Sci..

[69] Huang Y, Chubakov V, Mantovani F, Rudnick RL, McDonough WF (2013). A reference Earth model for the heat-producing elements and associated geoneutrino flux. Geochem. Geophys. Geosyst..

[70] Arcay D, Lallemand S, Doin MP (2008). Back-arc strain in subduction zones: Statistical observations versus numerical modeling. Geochem. Geophys. Geosyst..

[71] Espurt N (2008). Flat subduction dynamics and deformation of the South American plate: Insights from analog modeling. Tectonics.

[72] van Hunen J, van den Berg AP, Vlaar NJ (2004). Various mechanisms to induce present-day shallow flat subduction and implications for the younger Earth: A numerical parameter study. Phys. Earth Planet. Inter..

[73] Liu S, Currie CA (2016). Farallon plate dynamics prior to the Laramide orogeny: Numerical models of flat subduction. Tectonophysics.

[74] Manea VC, Marta PG, Manea M (2012). Chilean flat slab subduction controlled by overriding plate thickness and trench rollback. Geology.

[75] Manea V, Gurnis M (2007). Subduction zone evolution and low viscosity wedges and channels. Earth Planet. Sci. Lett..

[76] Rodríguez-González J, Negredo AM, Billen MI (2012). The role of the overriding plate thermal state on slab dip variability and on the occurrence of flat subduction. Geochem. Geophys. Geosyst..

[77] Shemenda A (1993). Subduction of the lithosphere and back arc dynamics: Insights from physical modeling. J. Geophys. Res..

[78] Schellart WP (2020). Control of subduction zone age and size on flat slab subduction. Front. Earth Sci. (Lausanne).

[79] Strak V, Schellart WP (2021). Thermo-mechanical numerical modeling of the South American subduction zone: A multi-parametric investigation. J. Geophys. Res. Solid Earth.

[80] Flórez-Rodríguez AG, Schellart WP, Strak V (2019). Impact of aseismic ridges on subduction systems: Insights from analog modeling. J. Geophys. Res. Solid Earth.

[81] Replumaz A, Funiciello F, Reitano R, Faccenna C, Balon M (2016). Asian collisional subduction: A key process driving formation of the Tibetan Plateau. Geology.

[82] Pitard P, Replumaz A, Funiciello F, Husson L, Faccenna C (2018). Mantle kinematics driving collisional subduction: Insights from analogue modeling. Earth Planet. Sci. Lett..

[83] Pusok AE, Kaus BJP (2015). Development of topography in 3-D continental-collision models. Geochem. Geophys. Geosyst..

[84] Dvorkin J, Nur A, Mavko G, Ben-Avraham Z (1993). Narrow subducting slabs and the origin of backarc basins. Tectonophysics.

